# A Novel Hypothetical Approach to Explain the Mechanisms of Pathogenicity of Rheumatic Arthritis

**DOI:** 10.31138/mjr.32.2.112

**Published:** 2021-05-28

**Authors:** Mark Feldman, Isaac Ginsburg

**Affiliations:** Institute for Dental Sciences, the Hebrew University – Hadassah Faculty of Dental Medicine, Ein Kerem Campus, Jerusalem, Israel

**Keywords:** Rheumatoid arthritis, ROS, RNS, proteinase, heparinoid, fibrinolysis, phospholipase

## Abstract

The autoimmune disorder rheumatoid arthritis (RA) is a relapsing and chronic inflammatory disease that affects the synovial cells, cartilage, bone, and muscle. It is characterised by the accumulation of huge numbers of polymorphonuclear neutrophils (PMNs) and macrophages in the synovia. Auto-antibodies are deposited in the joint via the activity of highly cationic histones released from neutrophil extracellular traps (NETs) in a phenomenon termed NETosis. The cationic histones function as opsonic agents that bind to negatively charged domains in autoantibodies and complement compounds via strong electrostatic forces, facilitating their deposition and endocytosis by synovial cells. However, eventually the main cause of tissue damage is the plethora of toxic pro-inflammatory substances released by activated neutrophils recruited by cytokines. Tissue damage in RA can also be accompanied by infections which, upon bacteriolysis, release cell-wall components that are toxic to tissues. Some amelioration of the damaged cells and tissues in RA may be achieved by the use of highly anionic heparins, which can neutralize cationic histone activity, provided that these polyanions are co-administrated with anti-inflammatory drugs such as steroids, colchicine, or methotrexate, low molecular weight antioxidants, proteinase inhibitors, and phospholipase A2 inhibitors.

## INTRODUCTION

Rheumatoid arthritis (RA) is a severe autoimmune disorder that occurs in about 0.5–1% of the population. It may lead to severe synovial, joint, cartilage and bone damage and dysfunction. Early diagnosis and treatment of RA can substantially slow the progression of joint damage in up to 90% of patients. Rheumatoid arthritis can also show systemic manifestations, such as cardiovascular, pulmonary, and skeletal disorders.^1–5^ However, the genetic environment that potentially leads to articular damage is still not fully understood.^6^

The hallmark of RA is deposition in the joints of rheumatoid factors, immune complexes, complement components^7–10^ and perivascular polymorphonuclear neutrophils (PMNs) and macrophage infiltration recruited via cytokines.^11–16^ These leukocytes become activated and can release a plethora of toxic pro-inflammatory agonists into the surroundings, which can synergistically attack and destroy synovial cells, cartilage and bone structures.

## A NOVEL WORKING HYPOTHESIS IS PROPOSED TO EXPLAIN MECHANISMS GOVERNING RA PATHOGENICITY

The present communication argues that highly cationic histones and additional polycations are delivered by neutrophil extracellular traps (NETs) in a process termed NETosis.^17–18^ These polycations act like opsonins19 that bind, via strong electrostatic forces, to negatively charged domains in immune complexes and in complement components. This interaction facilitates their deposition, binding and internalization by synovial cells.

This novel proposal is based mainly on previous observations from our laboratories. We have shown that if haemolytic streptococci, *Candida albicans*^20^ and even whole cell nuclei^21^ were pre-coated (opsonised) with the cationic histone, they will not only bind to and be endocytosed by phagocytes such as neutrophils and macrophages, but also by endothelial cells, fibroblasts, and even synovial cells.

However, the main toxic agents found in the joints that can eventually destroy synovial cells, cartilage and bone are most probably mediated by the toxic actions of the plethora of pro-inflammatory compounds released by activated neutrophils and macrophages. These include reactive oxygen and nitrogen species, cationic proteinases, and the membrane-damaging phospholipases and lysophosphatides,^22–36^ besides the toxic products released by dying cells.^37^ It is also of great interest to note that the mechanisms by which PMNs and macrophages destroy tissues are very similar to those mediated by group A haemolytic streptococci.^38^

## THE POSSIBLE ROLE PLAYED BY INFECTIONS IN RA PATHOGENICITY

Microbial infections, which sometimes accompany RA, may also contribute to the pathogenicity of various auto-immune disorders. This is because both pathogenic and non-pathogenic microorganisms can undergo bacteriolysis^39^ induced either by the action of cationic lysozyme,40 or following treatment with certain bacteriolytic antibiotics.^39^ Bacteriolysis involves the release of the cell-membrane component lipoteichoic acid and cell-wall-derived peptidoglycans^38^ derived from Gram-positive bacteria and lipopolysaccharide (LPS) from Gram-negative ones.^38^ Several studies have also described the persistence of non-biodegradable streptococcal cell-wall components in macrophages causing chronic joint inflammation.^41–45^ Moreover, during serious infections, treatment with anti-tumour necrosis factor alpha (TNFα) may also accompany RA.^46^

## ROLE OF REACTIVE OXYGEN SPECIES (ROS) IN RA PATHOGENICITY

Cationic proteinases and oxidants generated by activated leukocytes via NADPH oxidase, may be key products in tissue destruction during inflammation, and, most probably, also in autoimmune disorders.^47–58^ Therefore, the clinical use of low-molecular-weight antioxidants such as ascorbate, N-acetyl cysteine, glutathione, catalase, and antioxidant extracts from cranberries, lemon, and pomegranate. might be important in the early phases of RA, especially if also combined with the anti-proteinase aprotinin, and inhibitors of phospholipase A2 (PLA2) and lysophosphatides such as lecithin (see below).

## THE ROLE OF REACTIVE NITROGEN SPECIES (RNS) IN RA PATHOGENICITY

PMN activation in inflamed tissue can result in the generation of nitric oxide generated by inducible nitric oxide synthase. The nitric oxide radical can then be transformed into the highly toxic peroxynitrite which may act together with ROS to cause cell and tissue damage.^59–62^

## THE POSSIBLE ROLE OF PROTEINASES IN TISSUE DAMAGE IN RA

ROS, RNS and proteinases are always found in the synovial fluid of patients suffering from RA, and may be pathogenic, causing tissue degradation. A large series of investigations, mostly using tissue cultures, have demonstrated the synergistic toxic effects of proteinases and ROS.^19,21,22,24,25,29,30,33,63–65^ In general, oxidized proteins are more susceptible to degradation by proteinase.^65^

## CAN THE HIGHLY ANIONIC HEPARIN AND HEPARINOIDS BE USED TO AMELIORATE TISSUE DAMAGE IN RA?

To prevent the binding, deposition, and endocytosis of immune complexes and complement components in tissues mediated by the action of cationic opsonins,^19^ one might consider the administration to RA patients of the highly negatively charged heparin and heparinoids.^66–70^ These common drugs could neutralise the opsonic activity of the polycationic histone, thereby preventing the deposition of immune complexes and complement components in tissues. Heparinoids^69^ have also been found to prevent the binding of immune complexes containing nucleosomal antigens to the glomerular basement membrane, thereby delaying the onset of autoimmune nephritis, and heparin inhibits the proliferation of human RA synoviocytes through the nuclear factor (NF)-κB pathway.^70^

## ROLE OF BLOOD COAGULATION AND FIBRINOLYSIS IN RA PATHOGENICITY

The severe injury to synovial cells in the joints of RA patients may lead to blood-vessel damage. The accumulation of plasma and its coagulation and fibrinolytic components may affect the pathological processes involved in tissue destruction in the synovial cells, cartilage, muscle, and bone.^71–76^ Plasmin is a potent proteolytic enzyme that acts in concert with ROS, RNS, proteinases and membrane-perforating phospholipases released from activated PMNs to cause cell damage.^22,23^ Fibrinolysis may also be affected by polycations such as poly-L-lysine (a histone mimetic) and others.^77^

## ROLE OF PHOSPHOLIPASES IN RA PATHOGENICITY

### PLA2 activity in the serum and synovial fluids in RA

PLA2 released by activated PMNs is found in the sera and synovial fluids of RA patients, with a positive correlation between synovial fluid and matched sera.^78–82^ Human neutrophils that were pre-treated with subtoxic concentrations of PLA2-derived lysophosphatides acted synergistically with the neutrophil agonist phorbol myristate acetate (PMA), immune complexes, cationic poly-L-histidine, phytohemagglutinin, and N-formyl methionine-leucyl-phenylalanine (f-MLP) to cause enhanced generation of superoxide (O_2_^–^). The lysophosphatide compounds bind strongly to the neutrophils and could not be washed away. The lysophosphatides that collaborated with agonists to stimulate O_2_^–^ generation were also highly haemolytic towards human red blood cells. O_2_^–^ generation was also markedly enhanced when substimulatory amounts of arachidonic acid or eicosapentaenoic acid were added to PMNs in the presence of a variety of agonists.^83^ These data suggest that in addition to long-chain fatty acids, only those lysophosphatides compounds that possess fatty acids with more than 10 carbons and that are also highly haemolytic, can cause enhanced generation of O_2_^–^ in stimulated PMNs.

## ARE THERE ANY SPECIFIC CLINICAL DRUGS, OTHER THAN ANIONIC HEPARINS, WHICH CAN AMELIORATE TISSUE DAMAGE IN RA PATIENTS?

Inhibition of cell damage in RA has been attempted clinically using drugs such as the anti-inflammatories methotrexate, colchicine, steroids, and cyclophosphamides, all known suppressors of the PMNs’ main functions of chemotaxis and phagocytosis. Other suggested and tested drugs include hydroxychloroquine (Plaquenil), leflunomide (Arava), sulfasalazine (Azulfidine), and minocycline (Minocin).^84,85^ Chrysotherapy has also been recommended.^86^ However, to date, no drug has been really shown to effectively suppress the severe damage seen in RA. The use of antibiotics in RA may have some value when a specific microorganism is identified, but only if bacteriolytic antibiotics are used.^39^

## CONCLUSIONS, AND WHERE DO WE GO FROM HERE?

The present communication offers a novel approach to describe the possible mechanisms underlying the joint destruction that is a hallmark of RA pathogenicity. RA is a synergistic multifactorial autoimmune disease involving an interplay among a multiplicity of proinflammatory agonists generated by activated neutrophils and macrophages, reactive oxygen nitrogen species PLA2, lysophosphatides, autoimmune complexes, complement components, and highly positively charged histones generated by PMN NETosis. Unfortunately, no specific agents or agent combinations have been identified whose significant inhibition might alter the deleterious toxic effects leading to joint destruction.

## SUMMARY

In summary, we have discussed several overlapping and successive steps in the development and progression of RA pathogenicity. These steps include:

1. Recruitment of huge numbers of neutrophils (PMNs) and macrophages to the synovial area via cytokines.
2. Release from PMN NETs (NETosis) of highly cationic toxic histones and formation of citrullinated histones.
3. Histone may function as a potent opsonic agent.
4. Through strong electrostatic forces, opsonins can interact with and bind to negatively charged domains on immune complexes and complement components, facilitating their binding, deposition, and possibly also internalisation, by synovial cells.
5. Recruited PMNs and macrophages adhering to synovial surfaces are activated and release a plethora of toxic proinflammatory agonists into the surrounding medium. These include cationic peptides, oxidants, proteinases, and membrane-perforating phospholipases, which can all act synergistically to destroy synovial cells, cartilage, and bone structures. Development of blood coagulation and fibrinolysis in the synovial fluid may be dealt with using anticoagulants and agents that inhibit fibrinolytic activity.
6. Highly anionic heparin and heparinoids, which neutralise polycations, may provide protection against tissue damage in RA, more so if combined with drugs such as steroids, methotrexate, and colchicine, all potent anti-inflammatory agents against the PMN and macrophage functions of chemotaxis and phagocytosis. Th1 cytokines may also be inhibited by drugs that affect leukocyte recruitment.
7. Non-bacteriolytic antibiotics may be used to treat infections in RA patients. Bacteriolysis should be avoided, as it can release the potent toxic cell-wall components lipoteichoic acid, peptidoglycan, and endotoxin.
8. Nonbiodegradable microbial cell-wall components that persist for long periods in macrophages in the joints may perpetuate chronic destructive arthritis.
9. Toxic oxidants may be controlled clinically by the low-molecular-weight anti-oxidants glutathione, ascorbate, and N-acetyl cysteine, and by certain plant polyphenols.
10. Blood coagulation and fibrinolysis may be dealt with using the anticoagulant heparin and proteinase inhibitors.
11. The use of drug cocktails comprised of antioxidants, proteinase inhibitors, PLA2 inhibitors, highly anionic heparin to inhibit one of the major agonists-histones, is recommended, if also combined with drugs such as steroids. Clinically, this might be a complicated task, necessitating the use of appropriate animal models and the permission to use such cocktails clinically, which can only be obtained following highly expensive clinical trials in humans.
